# Pure ductal adenocarcinoma of the prostate protruding into the prostatic urethra: A case report of MRI findings and literature review

**DOI:** 10.1259/bjrcr.20210234

**Published:** 2022-09-12

**Authors:** Takahiro Yamamoto, Yumi Takehara, Sou Adachi, Akiko Narita, Natsuki Taniguchi, Kojiro Suzuki

**Affiliations:** 1 Department of Radiology, Aichi Medical University, 1-1 Yazako Karimata, Nagakute City, Aichi, Japan; 2 Department of Surgical Pathology, Aichi Medical University, 1-1 Yazako Karimata, Nagakute City, Aichi, Japan

## Abstract

Ductal adenocarcinoma of the prostate (DCa) is the histological variant of prostatic carcinoma. The macroscopic finding of DCa arising from primary duct by urethroscopy is papillary excrescences in the prostatic urethra. But the finding of MRI remains poorly understood, since there is no coherent report on the MRI finding of DCa arising from primary duct. We herein report a case of DCa arising from primary duct and forming papillary excrescences in the prostatic urethra. The patient was a male in his 70s and presented with gross hematuria a few days ago. Blood test showed elevated prostate specific antigen (PSA). Prostate MRI was performed. There were two lesions in the prostatic urethra and the right transition zone (TZ). On *T*_2_-weighted image (T2WI), the lesion in the prostatic urethra was identifiable, but the lesion in the right TZ was difficult to identify. On diffusion-weighted image (DWI), both lesions showed hyperintense signal and could be identified, and there was continuity between them. Urethroscopy was performed, there was the lesion with papillary excrescences developing from the right dorsal side of prostatic urethra. Transurethral resection of the prostate was performed. The pathological diagnosis was DCa (pure type). A review of previous literature showed that DCa had a slightly hypointense signal on T2WI. It may be difficult to identify DCa in the TZ because DCa and the TZ show similar signals on T2WI. DWI may be useful to accurately assess DCa arising from primary duct.

## Introduction

Ductal adenocarcinoma of the prostate (DCa) is the most common histological variant of prostatic carcinoma. The incidence of ductal adenocarcinoma is 3.2% of all prostatic carcinoma.^1^ DCa originates from the periurethral primary duct or from the more peripheral secondary duct. DCa arising from primary duct often protrude into the prostatic urethra at or near the verumontanum, forming friable gray-white papillary or polypoid excrescences that are visible urethroscopy and may create urethral obstructive and haematuria.^2^ In this study, we report our experience of a case of ductal adenocarcinoma of the prostate of primary duct origin with papillary excrescences within the prostatic urethra that could be identified by MRI, with some literature reviews.

## Clinical presentation

The patient was a male in his 70s. Gross hematuria appeared a few days ago. Since there was no improvement, the patient visited a department of urology in our hospital for further examination and treatment. Urinalysis was positive for occult blood in urine (2+). Urine cytology was negative. Blood test showed that there was no anaemia but elevated prostate-specific antigen (PSA) (5.83 ng ml^−1^; normal range,≤4.0 ng ml^−1^).

**Figure 1.**
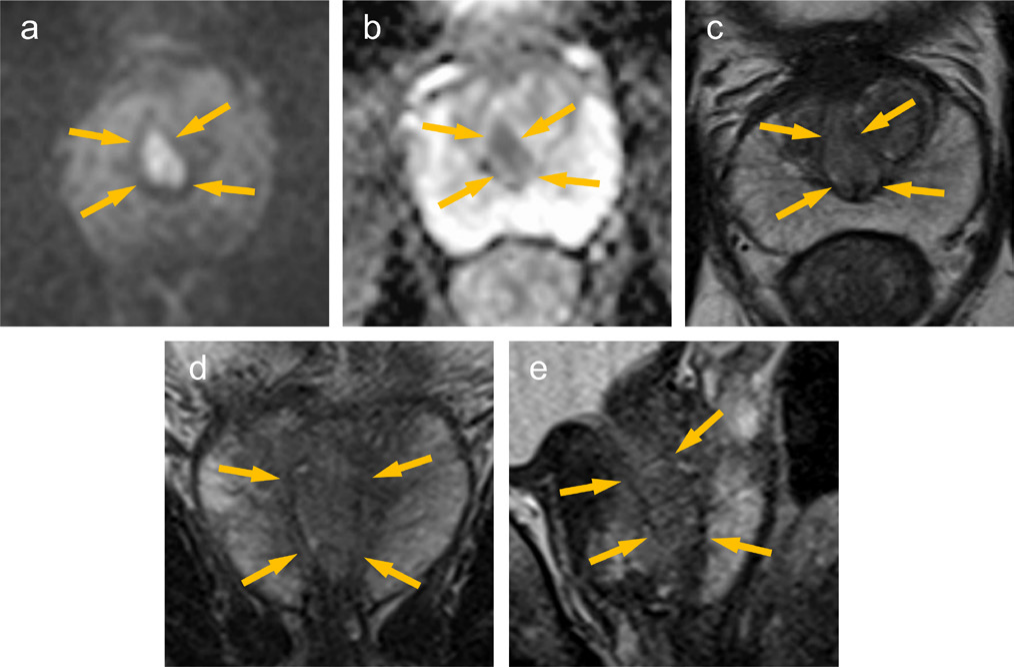
MRI (3.0T). There is an occupying lesion in the prostatic urethra (→). The lesion shows hyperintense signal on DWI (**a**), hypointense signal on ADCmap (**b**). On T2WI, the lesion shows slightly inhomogeneous hypointense signal similar to hypertrophic nodule in the transition zone (c; axial image, d; coronal image, e; sagittal image).

**Figure 2.**
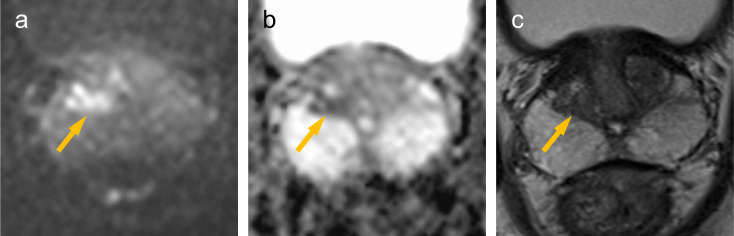
MRI (3.0T). There is a nodule in the right transition zone (→). The nodule shows hyperintense signal on DWI (**a**), hypointense signal on ADCmap (**b**). But the nodule is not identifiable on T2WI, since the nodule shows equal signal to the surrounding transition zone (**c**).

**Figure 3.**
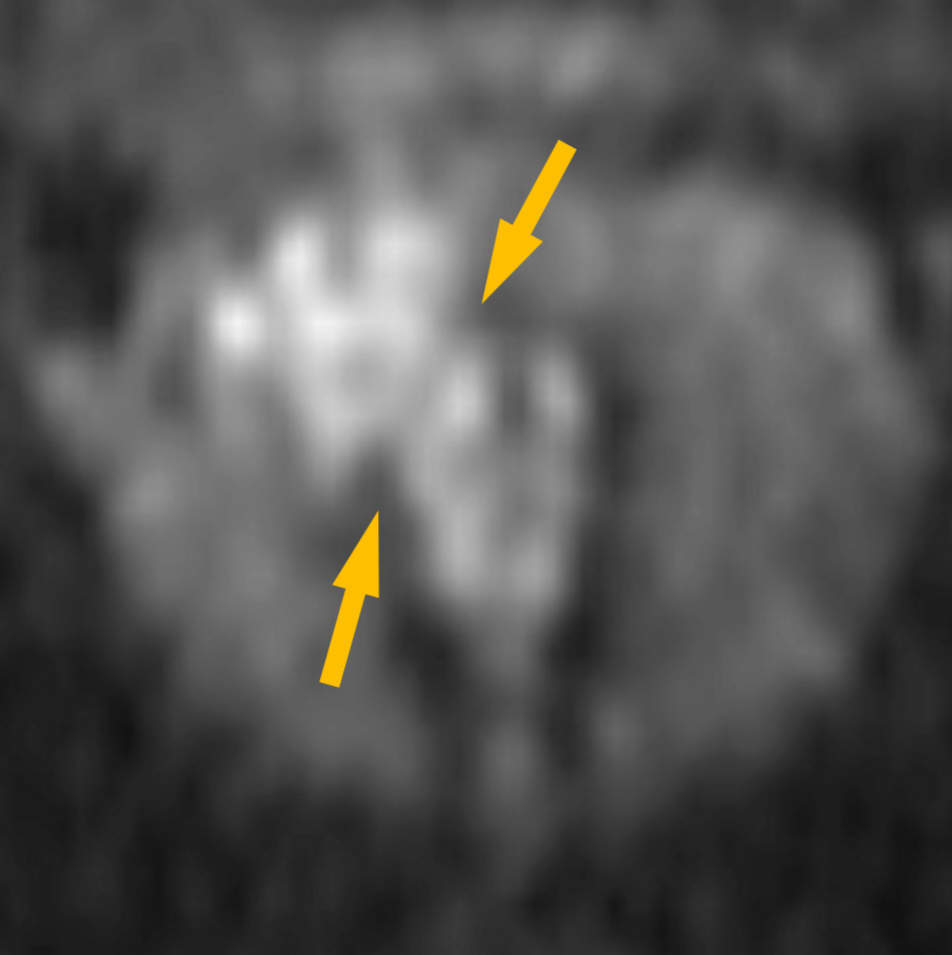
MPR coronal image with the prostatic urethra as the axis on DWI. The continuity between the lesion in the prostatic urethra and the nodule in the right transition zone became clearer (→).

Prostate MRI (3.0T) was performed according to Prostate Imaging Reporting and Data System (PI-RADS) ver2.1.^3^ Dynamic contrast-enhanced MRI was not performed at this time, only unenhanced MRI was performed. Prostate was 5× 5.4 cm×3.7 cm and PSA density was 0.11 (ng/mL/cm^3^). The prostatic urethra was dilated, with an occupying lesion inside. The lesion was 9 × 12 × 23 mm on *T*_2_-weighted image (T2WI). The lesion showed hyperintense signal on diffusion-weighted image (DWI), hypointense signal on apparent diffusion coefficient (ADC) map, and slightly inhomogeneous hypointense signal similar to hypertrophic nodule in the transition zone (TZ) on T2WI (Figure 1). There was a nodule in the right TZ (Figure 2). The nodule was 11 ×  6 × 4 mm on DWI, showing hyperintense signal on DWI and hypointense signal on ADCmap. But the nodule was not identifiable on T2WI, since the nodule showed equal signal to the surrounding TZ. The result showed that the PI-RADS category for the lesion in the TZ was 3 (T2WI score 3, DWI score 4). On DWI, the boundary between the lesion in the prostatic urethra and the TZ was clear, but only the area bordering the nodule in the right dorsal TZ was slightly unclear. There was a suspected continuity between the lesion in the prostatic urethra and the nodule in the right dorsal TZ on DWI. Multiplaner reconstruction (MPR) image was created with the prostatic urethra as the axis on DWI. On MPR coronal image, the continuity between the lesion in the prostatic urethra and the nodule in the right TZ became clearer (Figure 3). Chest and abdominopelvic CT was performed, there were no findings of lymph node metastasis or distant metastasis. Urothelial carcinoma, conventional acinar adenocarcinoma of the prostate and ductal adenocarcinoma of the prostate were considered as differential diseases.

**Figure 4.**
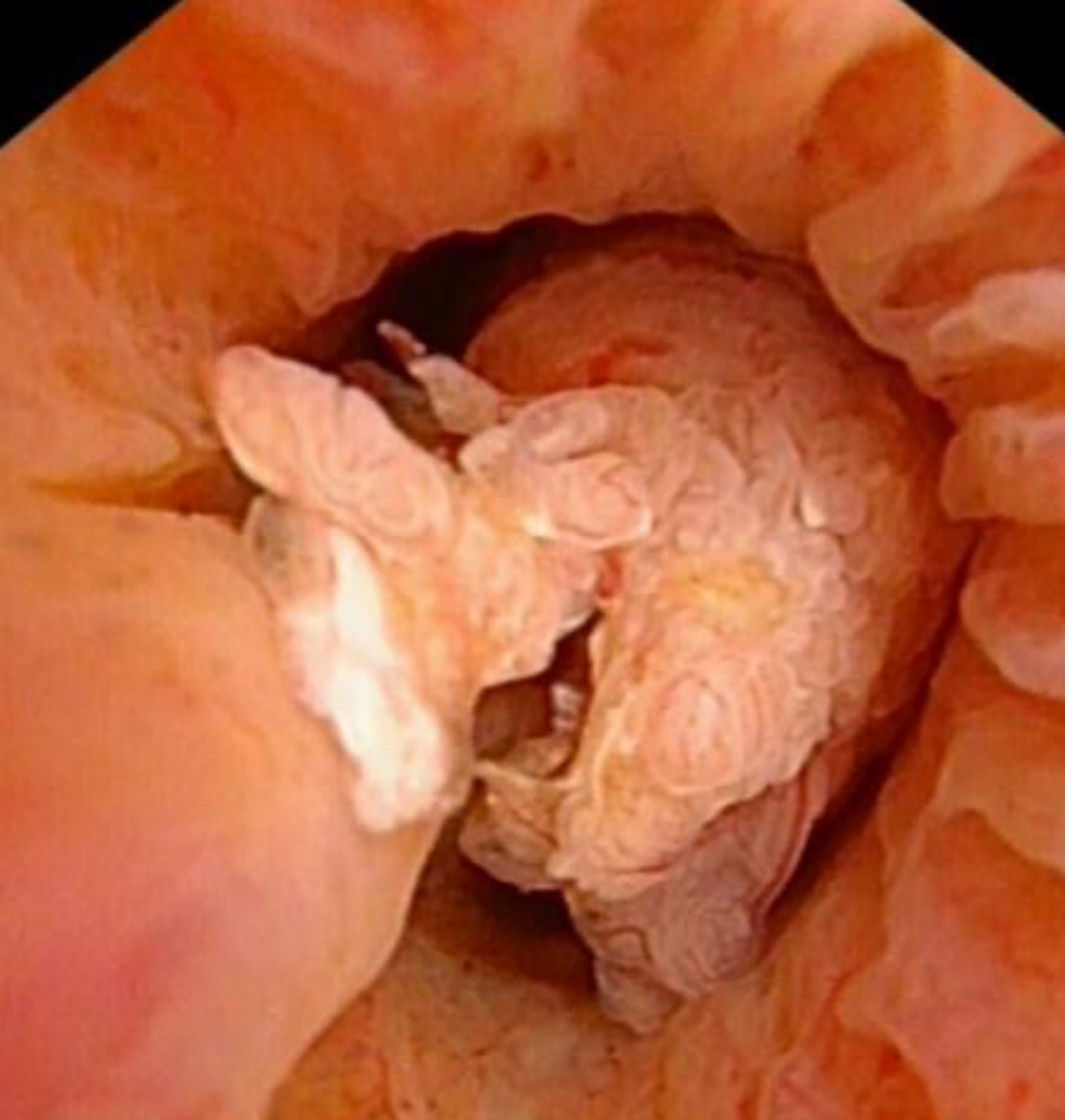
Urethroscopy image. The lesion with a papillary appearance arises from the right dorsal side-of prostatic urethra.

**Figure 5.**
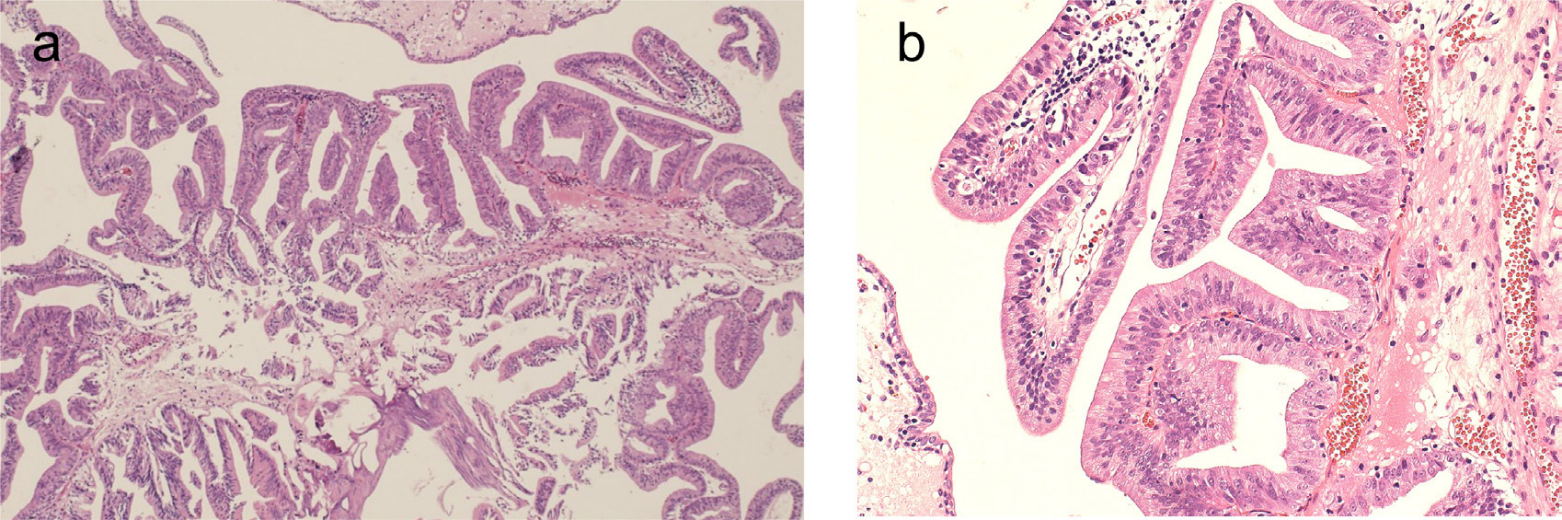
Hematoxylin and eosin staining. Atypical cells proliferate in a papillary pattern (**a**), and pseudostratified columnar epithelium are found (**b**).

Urethroscopy was performed, there was the lesion with papillary excrescences developing from the right dorsal side of prostatic urethra (Figure 4). Transurethral resection of the prostate was performed. Microscopically, atypical cells proliferating in a papillary pattern with pseudostratified columnar epithelium were found (Figure 5). Immunostaining was positive for NKX3.1 and AMACR, supporting that the tumor was of prostate origin. Since no component of acinar adenocarcinoma was found, so the diagnosis of ductal adenocarcinoma of the prostate (pure type) was confirmed. Since there was a possibility of residual tumor, hormone therapy and radiation therapy (74 Gy/ 34 fractions) were administered. After the therapy, the PSA decreased to 0.01 ng ml^−1^, and the patient has uneventful course without recurrence for about 2 years.

## Discussion

Ductal adenocarcinoma of the prostate (DCa) is a histological variant of prostatic carcinoma. Microscopically, DCa is characterized by pseudostratified columnar epithelium.^1^ There are two types of ductal adenocarcinoma: mixed type, which includes acinar adenocarcinoma, and pure type, which does not. Mixed ductal adenocarcinoma is more common than pure ductal adenocarsinoma.^1^ Our case was considered to be pure type, because no acinar adenocarcinoma was found in the pathological diagnosis. DCa is more prone to distant metastasis and has a lower elevation of PSA level than conventional acinar adenocarcinoma of the prostate (CCa).^4^

On MRI, DCa shows hyperintense signal on DWI, hypointense signal on ADCmap and T2WI, just like CCa. However, there are some differences between DCa and CCa. DCa shows a strong hyperintense signal on DWI, similar to that of high (Gleason score≥7) grade CCa, although a slightly hypointense signal on T2WI, similar to that of low (Gleason score 3 + 3=6) grade CCa.^5^ DCa resembles low grade CCa on T2WI, which underestimates tumour grade and renders the tumour occult.^6^ In previous reports Coffey N et al and Schieda N et al reported imaging findings of MRI in 8 and 11 cases of DCa, respectively, but only 2 cases included DCa located in the transition zone (TZ).^4. 5^ Therefore, the details of MRI findings of the DCa located in the TZ have not been revealed. In our case, it was difficult to identify the part of DCa in the TZ on T2WI, since the lesion showed equal signal to the surrounding TZ. The slightly hypointense signal on T2WI of DCa can be identified in the peripheral zone (PZ) showing hyperintense signal. It may be difficult to be identify DCa in the TZ showing slightly hypointense signal. In contrast, the part of DCa in the TZ was clearly identifiable on DWI and ADCmap, since the lesion showed clearly hyperintense signal on DWI and hypointense signal on ADCmap.

DCa arising from primary duct often characteristically present with papillary excrescences in the prostatic urethra on urethroscopy.^1^ However, the finding of MRI remains poorly understood, since there is no coherent report on the MRI findings of DCa arising from primary duct with papillary excrescences in the prostatic urethra. In our case, the papillary excrescences in the prostatic urethra were identifiable on MRI, showing hyperintense signal on DWI, similar to that of the lesion in the TZ, and slightly inhomogeneous hypointense signal similar to hypertrophic nodule in the TZ onT2WI.

The continuity between the part of DCa in the TZ and those in in the prostatic urethra was not clear onT2WI because the part of DCa in the TZ could not be identified. On DWI, both the part of DCa in the TZ and those in the prostatic urethra showed hyperintense signal and could be identified. MPR coronal image with the prostatic urethra as the axis on DWI showed a clearer continuity between the part of DCa in the prostatic urethra and those in the TZ. This MRI findings was consistent with the urethroscopic findings.

Ductal adenocarcinoma of the prostate (DCa) arising from primary duct often protrude into the prostatic urethra, forming papillary excrescences. In our case, on T2WI, the papillary excrescences in the prostatic urethra could be identified, but the lesion within the TZ could not be identified. DWI were useful to evaluate the entire lesion. MPR images with the prostatic urethra as the axis on DWI may further aid in assessing the continuity between the part of DCa in the prostatic urethra and those in the TZ.

## Learning points

- Ductal adenocarcinoma of the prostate arising from primary duct often protrude into the prostatic urethra with forming papillary excrescences.
- It may be difficult to identify the part of prostatic ductal adenocarcinoma in the transition zone on T2WI.
- To evaluate the continuity between the ductal adenocarcinoma of the prostate in the prostatic urethra and those in the transition zone, it may be useful to create MPR images with the prostatic urethra as the axis on DWI.
